# The People with Asperger syndrome and anxiety disorders (PAsSA) trial: a pilot multicentre, single-blind randomised trial of group cognitive–behavioural therapy

**DOI:** 10.1192/bjpo.bp.115.002527

**Published:** 2016-04-13

**Authors:** Peter E. Langdon, Glynis H. Murphy, Lee Shepstone, Edward C.F. Wilson, David Fowler, David Heavens, Aida Malovic, Alexandra Russell, Alice Rose, Louise Mullineaux

**Affiliations:** **Peter E. Langdon**, DClinPsy, PhD, Tizard Centre, University of Kent, Canterbury, UK; Broadland Clinic, Hertfordshire Partnership University NHS Foundation Trust in Norfolk, Norwich, UK; **Glynis H. Murphy**, PhD, Tizard Centre, University of Kent, Canterbury, UK, and Oxleas NHS Foundation Trust, Dartford, UK; **Lee Shepstone**, PhD, Department of Population Health and Primary Care, Norwich Medical School, University of East Anglia, Norwich, UK; **Edward C.F. Wilson**, PhD, School of Clinical Medicine, University of Cambridge, Cambridge, UK; **David Fowler**, MSc, School of Psychology, University of Sussex, Brighton, UK; **David Heavens**, ClinPsyD, Department of Clinical Psychology, Norwich Medical School, University of East Anglia, Norwich, and Norfolk and Suffolk NHS Foundation Trust, Norwich, UK; **Alexandra Russell**, BSc (Hons); **Alice Rose**, BSc (Hons), Department of Clinical Psychology, Norwich Medical School, University of East Anglia, Norwich, UK, and Norfolk and Suffolk NHS Foundation Trust, Norwich, UK; **Aida Malovic**, MSc, Tizard Centre, University of Kent, Canterbury, UK; **Louise Mullineaux**, BSc (Hons), Department of Clinical Psychology, Norwich Medical School, University of East Anglia, Norwich, UK, and Hertfordshire Partnership University NHS Foundation Trust in Norfolk, Norwich, UK

## Abstract

**Background:**

There is a growing interest in using cognitive–behavioural therapy (CBT) with people who have Asperger syndrome and comorbid mental health problems.

**Aims:**

To examine whether modified group CBT for clinically significant anxiety in an Asperger syndrome population is feasible and likely to be efficacious.

**Method:**

Using a randomised assessor-blind trial, 52 individuals with Asperger syndrome were randomised into a treatment arm or a waiting-list control arm. After 24 weeks, those in the waiting-list control arm received treatment, while those initially randomised to treatment were followed up for 24 weeks.

**Results:**

The conversion rate for this trial was high (1.6:1), while attrition was 13%. After 24 weeks, there was no significant difference between those randomised to the treatment arm compared with those randomised to the waiting-list control arm on the primary outcome measure, the Hamilton Rating Scale for Anxiety.

**Conclusions:**

Trials of psychological therapies with this population are feasible. Larger definitive trials are now needed.

**Declaration of interest:**

None.

**Copyright and usage:**

© The Royal College of Psychiatrists 2016. This is an open access article distributed under the terms of the Creative Commons Attribution (CC BY) licence.

Anxiety disorders and related symptomatology are commonly found among those with autistic spectrum disorders (ASDs), including Asperger syndrome.^1–7^ A meta-analysis examining the effectiveness of cognitive–behavioural therapy (CBT) for anxiety disorders in children with ASDs reported that treatment had an effect size of *d*=1.19 for clinician-rated outcome measures, *d*=1.21 for parent-rated outcome measures and *d*=0.68 for child self-report outcome measures.^8^ The literature about the treatment of mental health problems for adults with ASDs using CBT remains relatively sparse; there have been some case studies^9,10^ and some small trials.^11–14^ The aim of this trial was to collect data sufficient to inform the design of a definitive large-scale trial. The specific objectives included (a) assessing whether a CBT intervention is likely to be efficacious within a pilot, assessor-blind RCT with adults who have Asperger syndrome experiencing problems with anxiety, and (b) to gain participant views about taking part in therapy.

## Method

### Participants

**Fig. 1:**
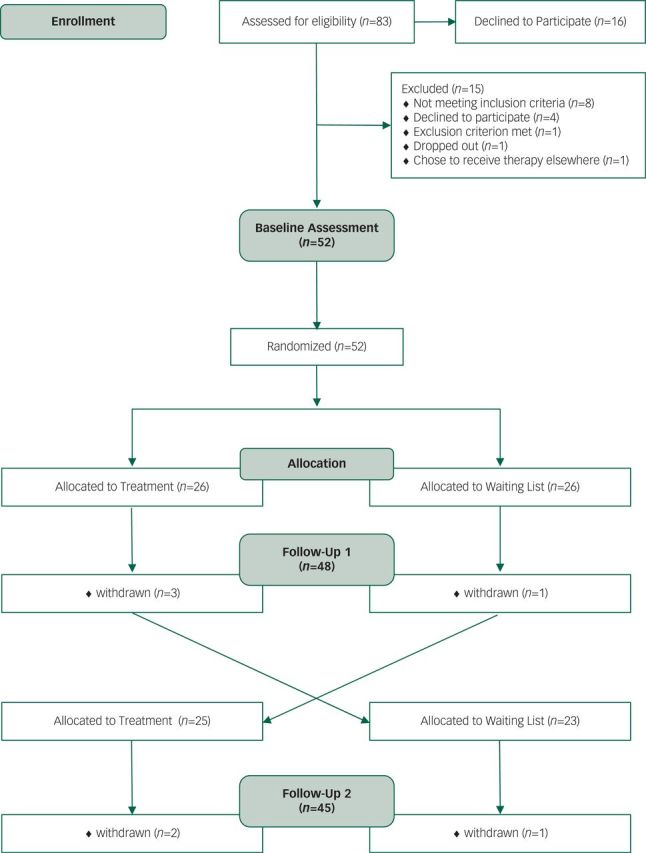
Consort diagram depicting participant flow throughout the trial.

Fifty-two individuals, *M*_age_=35.9, s.d.=14.5, 48% women, were recruited and enrolled within the trial from Kent, South East London and Norfolk within the UK. Recruitment took place within the community from Asperger syndrome/autism teams, Asperger syndrome user groups, such as Asperger East Anglia, the Kent Autistic Trust, Bridging the Gap, the Disability and Dyslexia Support Services at the University of Kent, intellectual disability teams, adult mental health teams, and through public advertisements. Participant flow throughout the trial is found in Fig. 1, and further demographic information can be found in Table 1.

**Table 1:** Baseline characteristics

	Group		
	
	Treatment arm (*n*=26)	Waiting list (*n*=26)	Combined (*n*=52)
Age, years: mean (s.d.) range	33.1 (14.6) 20–64	38.7 (14.3) 17–65	35.9 (14.6) 17–65

Male, *n* (%)	12 (46)	15 (58)	27 (52)
Female, *n* (%)	14 (54)	11 (42)	25 (48)

IQ: mean (s.d.) range	106.18 (17.14) 71.00–135.00	104.83 (11.51) 74.00–128.00	105.51 (14.33) 71.00–135.00

Ethnicity, *n* (%)			
White British	25 (96)	26 (100)	51 (98)
White other	1 (4)	0	1 (2)

Marital status, *n* (%)			
Single	19 (73%)	17 (65)	36 (69)
Cohabiting	0	2 (8)	2 (4)
Married	4 (15%)	5 (19)	9 (17)
Divorced	3 (12%)	2 (8)	5 (10)

Children, *n* (%)			
None	20 (77)	20 (77)	40 (77)
1	0	2 (8)	2 (4)
2	2 (8)	3 (12)	5 (10)
3	4 (15)	0	4 (8)
>3	0	1 (4)	1 (2)

Education			
Primary or less	1 (4)	0	1 (2)
Secondary	7 (27)	9 (35)	16 (31)
Tertiary	8 (31)	9 (35)	17 (33)
University degree	10 (38)	8 (31)	18 (35)

All participants were initially screened by research workers to determine eligibility to take part in the trial. The inclusion criteria were as follows: (a) participants fulfilled diagnostic criteria for Asperger syndrome, high-functioning autism or pervasive developmental disorder – not specified; diagnosis was confirmed by inspection of previous records, or the study team used the Autism Diagnostic Observation Schedule to confirm the diagnosis; (b) participants had clinically significant difficulties with anxiety as confirmed through the use of the Hamilton Rating Scale for Anxiety (HAM-A; score of >14 qualified inclusion); (c) participants were between 16 and 65 years of age; (d) full Scale IQ>70 on the Wechsler Abbreviated Scale of Intelligence.^15^ The exclusion criteria were as follows: (a) participants with post-traumatic stress disorder, or anxiety related to substance misuse; (b) comorbid severe psychiatric disorders that impair capacity to consent to take part (e.g. florid symptoms of psychosis); and (c) current substance misuse such as alcohol or drugs.

### Design and randomisation

This study was an assessor-blind randomised trial. Our full protocol has been published elsewhere.^16^ Masked researchers enrolled participants and carried out the assessments. Even pairs of participants were allocated to the treatment arm (group CBT+ treatment as usual (TAU) for 24 weeks) or the waiting-list control arm (TAU for 24 weeks) using blocked randomisation with random even blocks, stratified by study site. The therapists at each research site contacted participants to inform them of their group allocation. All data were stored independently by the Norwich Clinical Trials Unit based at the University of East Anglia, who also carried out randomisation. Once randomisation had been completed, therapists were informed, who then informed participants.

After the initial 24 weeks of treatment, those within the waiting-list control group received 24 weeks of group CBT, while those who had already taken part in group CBT continued to receive TAU for a further 24 weeks. Both groups were assessed again following this further 24-week period. This allowed for 50% of the participants, those who received treatment first, to be followed up for a 6-month period. Following the completion of all study outcome measures, participants were interviewed and invited to give their opinions on the intervention and asked to make suggestions about what they would like to change. Participants completed the outcome measures on three occasions: (a) baseline, (b) follow-up 1 and (c) follow-up 2.

### Ethical considerations

A favourable ethical opinion was obtained from the Cambridgeshire 4 NHS Research Ethics Committee (ref.: 10/H0305/42). Informed consent was sought from participants and their carers, who completed the Social/Emotional Functioning Interview – Informant Version. Participants were afforded time to consider whether they wanted to participate and were given the opportunity to ask any questions. Information about the study was provided in an ‘easier to read’ format for participants who may have had reading difficulties. Participants were told that they could withdraw from the trial at any stage without giving a reason, and this would not affect access to other treatments or services. Adverse events relating to the trial were monitored throughout and none was detected.

### Intervention

The intervention used within this study comprised 24 weekly sessions, each lasting approximately 1 h. Participants received three initial sessions of 1:1 CBT, followed by 21 group CBT sessions. All sessions took placed within community-based settings. The initial three sessions of therapy aimed to help socialise each participant into CBT and to address any concerns they may have about joining the group. At least two therapists were present for each group session. In order to ensure adherence to treatment, a treatment manual^17^ was developed with specific aims for each session, and all sessions were delivered by a registered clinical psychologist or a qualified cognitive–behavioural therapist. The treatment manual included the following topics: (a) psychoeducation about ASDs and anxiety, (b) cognitive restructuring, (c) anxiety management techniques, (d) systematic desensitisation, (e) exposure to feared social situations and (f) social skills training. These skills were practiced *in vivo*. In addition to the intervention, participants in both arms before and after crossover received TAU. The description of our intervention is intentionally brief within this paper as the complete treatment manual, which includes all session-by-session contents, can be downloaded from http://www.kent.ac.uk/tizard/staff/acadstaff/pete_langdon.html.

### Outcome measures and analysis

Our primary outcome measure was the HAM-A. Data were collected at participants’ homes, the university or in community-based clinical settings.

#### Primary outcome measure

*1. Hamilton Rating Scale for Anxiety*. This is a structured clinician-rated scale incorporating 14 factors which are considered valid indicators of anxiety.^18^ Each factor reflects a symptom of anxiety; physical as well as mental symptoms are represented. The factors are scored on a 5-point scale as part of a structured interview.

#### Secondary outcome measures

*1. Social Phobia Inventory*. This is a 17-item self-report measure of behavioural, physiological and cognitive symptoms associated with social phobia.^19^ Participants rate the frequency with which they experience each symptom over the last week, using a 5-point Likert-type scale (0–4).

*2. Liebowitz Social Anxiety Scale.* This instrument is a self-report scale that assesses fear and avoidance throughout 24 listed situations, which are likely to elicit social anxiety.^20^

*3. Social and Emotional Functioning Interview (Informant and Subject Versions).* This is a semi-structured clinician-rated assessment of everyday social and psychiatric functioning that was designed to assess independence, leisure, interpersonal problems, employment and social relationships.^21^ Some items are shared with the Autistic Diagnostic Observation Schedule. This measure was completed with each participant and a nominated informant.

*4. Social Interaction Anxiety Scale*. A self-report 20-item measure of anxiety as experienced in social situations associated with social anxiety and social phobia in accordance to the DSM-IV criteria.^22,23^ Experiences are rated on a 5-point scale from 0 (not at all characteristic of me) to 4 (extremely characteristic of me).

*5. Fear Questionnaire.* A self-report questionnaire regarding the individual perception of fears and phobias; respondents are asked how likely they are to avoid each of the listed situations, due to anxiety/fear or any other unpleasant feelings.^24^ In addition to the 15 pre-existing items the individual is asked to document and score any individual phobias they would wish treated.

*6. Hamilton Rating Scale for Depression.* This structured clinician-rated interview is considered a valid indicator of depression and the ratings are based on the interviewer’s objective and subjective perceptions during the assessment.^25^ Eight items are scored on a 0 (not present) to 4 (severe) point scale, and nine items are scored from 0 to 2 (levels of severity).

##### Views about therapy

Following the completion of the trial, participants were interviewed, and asked to rate nine questions on a 5-point Likert Scale about their experiences of receiving therapy. Participants were also asked the five following questions, with supplementary questions used for clarification: (1) ‘What were you hoping for by taking part in this research study?’, (2) ‘What was best about the group?’, (3) ‘What was worst about the group?’, (4) ‘What advice would you give for the next group?’ and (5) ‘Were there any difficulties you feel that the group did not address?’.

##### Health economics

Generic health-related quality of life (EuroQol EQ-5D) and health service contact data were also collected. These will be reported separately (manuscript in preparation) and includes some description of TAU.

#### Analysis

Data were analysed using SAS Version 9.4 by a statistician (L.S.) masked to subgroup, controlling for baseline scores, making use of the intention-to-treat principle; the analysis was completed using the originally assigned groups. The initial group allocation for participants did not change throughout the trial. Data about participants’ views of taking part in therapy were subjected to both a frequency and descriptive thematic analysis. A supplementary analysis was completed using participants who attended at least 50% of the treatment sessions at follow-up 1 in order to examine whether outcomes were different for those who attended a greater number of sessions.

## Results

Considering recruitment, 83 participants were approached and 52 were enrolled; this is a conversion rate of 1.6 participants to 1 participant. In the course of the trial, seven participants were lost, representing an attrition rate of 13%. One participant told us that they withdrew because they found travelling to the group too difficult. Another three participants said that they no longer wanted to attend the group because it was either too difficult for them or something they found unhelpful. The other participants did not respond to our attempts to contact them.

The two treatment arms were well matched on IQ, age, gender and the primary outcome measure (Tables 1 and 2). Both groups scored in the ‘mild to moderate’ or ‘moderate to severe’ ranges on the HAM-A. Participants were predominately White British, single and without children (Table 1). The two groups did not differ significantly in terms of the number of treatment sessions they attended, *t* (50)≤1, *P*=0.774 (Table 2).

**Table 2:** Mean session attendance and primary outcome measure – Hamilton Rating Scale for Anxiety

	Group			
			
	Treatment arm (*n*=26), mean (s.d.)	Waiting list (*n*=26), mean (s.d.)	Mean difference (95% CI), *P*	Adjusted mean difference^a^ (95% CI), *P*
Session attendance	13.3 (7.17)	13.9 (7.27)	−0.58, (−3.4 to 4.6), 0.774	–

Hamilton Rating Scale for Anxiety				
Screening	27.2 (11.23) *n*=26	25.3 (13.92) *n*=26		–
Baseline	25.7 (11.99) *n*=26	22.8 (9.45) *n*=26		–
Follow-up 1	15.5 (7.91) *n*=23	16.3 (7.54) *n*=25	−0.84 (−5.3 to 3.6), 0.708	−2.46, (−5.9 to 1.0), 0.161
Follow-up 2	13.3 (8.57) *n*=22	13.6 (5.35) *n*=23	−0.29 (−4.6 to 4.0), 0.892	−0.91, (−5.0 to 3.2), 0.659

a Resulting from an ANCOVA including baseline score.

Turning to consider the primary outcome measure, HAM-A mean scores significantly improved over time, regardless of arm, and regardless of baseline scores, *F*(2,84)=43.67, *P*<0.001. Controlling for baseline scores, there was no significant difference between the treatment and wait list arms at either follow-up 1 or 2 on the HAM-A (Table 2).

Considering the secondary outcome measures, there was a significant improvement over time, regardless of arm, and baseline scores, on the Hamilton Rating Scale for Depression (HAM-D), *F*(2,84)=7.84, *P*=0.008; Fear Questionnaire Total Phobia Score, *F*(2,84)=6.00, *P*=0.019; Liebowitz Avoidance, *F*(2,84)=10.52, *P*=0.003; Liebowitz Fear/Anxiety, *F*(2,84)=10.90, *P*<0.002; Social Interaction Anxiety Scale, *F*(2,84)=16.75, *P*<0.001; Social Phobia Inventory, *F*(2,84)=8.15, *P*=0.007; the Social/Emotional Functioning Interview – Informant, *F*(2,84)=30.87, *P*<0.001; and Subject Versions, *F*(2,84)=17.37, *P*<0.001 (Table 3). Controlling for baseline scores, there was no significant difference between the treatment and wait list arms on any of the secondary outcomes at follow-up 1 or 2 (Table 3).

**Table 3:** Secondary outcome measures: means (standard deviations)

		Group		
			
		Treatment	Waiting list	Adjusted mean difference^a^ (95% CI), *P*
Hamilton Rating Scale – Depression	Baseline	20.5 (9.51)	17.5 (8.56)	
	Follow-up 1	17.5 (8.08)	17.2 (6.61)	−1.42, (−4.7 to 1.9), 0.396
	Follow-up 2	16.5 (9.68)	13.6 (5.39)	2.09, (−2.4 to 6.6), 0.353

Fear Questionnaire – Total Phobia	Baseline	50.9 (20.71)	42.3 (17.48)	
	Follow-up 1	43.2 (19.20)	36.1 (20.73)	−0.66, (−10.1 to 8.8), 0.890
	Follow-up 2	43.7 (22.87)	33.4 (21.54)	6.18, (−6.6 to 18.9), 0.338

Fear Questionnaire – Avoidance	Baseline	6.1 (2.40)	4.2 (3.16)	
	Follow-up 1	5.5 (2.79)	4.6 2.90)	1.68, (−0.0 to 3.4), 0.052
	Follow-up 2	5.1 (2.41)	3.5 (2.91)	0.13, (−1.7 to 2.0), 0.890

Fear Questionnaire – Anxiety/Depression	Baseline	23.2 (10.03)	20.7 (9.60)	
	Follow-up 1	21.4 (8.58)	19.1 (9.98)	0.06, (−4.0 to 4.1), 0.977
	Follow-up 2	18.4 (9.75)	16.5 (9.25)	1.21, (−4.2 to 6.6), 0.657

Fear Questionnaire – Global Rating	Baseline	5.2 (2.23)	4.9 (1.97)	
	Follow-up 1	3.0 (2.70)	3.8 (2.39)	−1.11, (−2.4 to 0.2), 0.094
	Follow-up 2	3.5 (2.60)	3.2 (1.92)	0.02, (−1.2 to 1.3), 0.980

Liebowitz avoidance	Baseline	42.2 (14.81)	40.3 (13.94)	
	Follow-up 1	39.2 (14.31)	34.1 (15.77)	1.47, (−5.0 to 8.0), 0.652
	Follow-up 2	34.5 (17.61)	28.8 (12.42)	6.40, (−1.5 to 14.3), 0.114

Liebowitz Fear/Anxiety	Baseline	43.4 (15.10)	43.4 (13.99)	
	Follow-up 1	42.2 (12.81)	36.8 (15.66)	3.09, (−2.2 to 9.0), 0.299
	Follow-up 2	39.1 (16.33)	31.6 (13.72)	7.33, (−0.8 to 15.5), 0.080

Social Interaction Anxiety Scale	Baseline	43.9 (13.56)	42.3 (13.53)	
	Follow-up 1	41.5 (14.08)	39.8 (16.59)	0.02, (−5.0 to 5.1), 0.994
	Follow-up 2	39.8 (14.65)	35.6 (13.22)	3.01, (−3.9 to 9.9), 0.390

Social Phobia Inventory	Baseline	34.3 (16.57)	31.6 (16.94)	
	Follow-up 1	33.0 (14.08)	25.4 (15.84)	4.69, (−1.7 to 11.0), 0.147
	Follow-up 2	27.2 (16.20)	24.3 (14.74)	1.99, (−5.9 to 9.9), 0.616

Social/Emotional Functioning Interview – Informant Version – Total Score	Baseline	57.5 (17.30)	53.8 (17.58)	
	Follow-up 1	52.5 (18.52)	51.2 (15.97)	−3.22, (−83 to 1.9), 0.210
	Follow-up 2	45.6 (16.16)	39.8 (14.78)	3.32, (−3.7 to 10.3), 0.343

Social/Emotional Functioning Interview – Subject Version – Total Score	Baseline	48.2 (21.22)	47.1 (23.71)	
	Follow-up 1	46.0 (18.94)	42.7 (14.41)	1.78, (−3.2 to 6.8), 0.478
	Follow-up 2	38.1 (18.00)	31.7 (12.28)	5.96, (−2.9 to 14.8), 0.183

a Resulting from an ANCOVA including baseline score.

Just over half (53%) of the participants agreed or strongly agreed that the individual sessions that were initially offered helped prepare them for the group sessions. It was also the case that over half (59%) of the participants agreed or strongly agreed that they now knew how to reduce their feelings of anxiety following treatment. However, 38% of participants thought there was insufficient time during sessions and 41% thought there were too few sessions. Seventy-nine per cent of participants agreed or strongly agreed that they found listening to the problems of others helpful, while nearly 80% agreed or strongly agreed that they felt supported by other group members. Just over half (56%) agreed or strongly agreed that therapy reduced their anxiety, while 44% were neutral, disagreed, or strongly disagreed on this. Seventy-three per cent of participants agreed or strongly agreed that they would recommend therapy to others, and 73% agreed or strongly agreed that therapy was helpful (Table 4).

**Table 4:** Participants responses to the questionnaire about experiences of receiving therapy

Question	Strongly agree (%)	Agree (%)	Neutral (%)	Disagree (%)	Strongly disagree (%)
The individual therapy prepared me for the group therapy.	26.5	26.5	35	12	–
Since attending, I now know what I can do to help reduce my anxious feelings.	21	38	23.5	12	6
There was sufficient time in sessions for my problems to be addressed.	26.5	21	15	38	–
There were enough sessions for my needs.	18	35	6	38	3
Listening to other group members talking about their problems was useful to me.	41	38	15	6	–
I felt supported by the other group members during the sessions.	26.5	53	12	9	–
I think the therapy has improved my anxiety.	18	38	32	6	6
I would recommend the therapy to others.	41	32	15	9	3
Overall, the therapy was helpful to me.	29	44	15	9	3

Turning to consider the open-ended questions that participants were asked at the end of the trial about their experience of taking part in therapy, five clear themes emerged which are largely framed around the questions asked. The first was labelled, ‘motivation to take part’. Participants described taking part in the trial in order to access help for their mental health problems, while others had hoped that they might form new relationships with other people with ASDs. Many told us that they wanted to ‘change their life for the better’ and recognised that anxiety was having a detrimental effect upon their well-being and ability to manage their lives.

The second theme was labelled, ‘positive experiences’. Participants described that they enjoyed ‘interacting with the others; meant a lot because we could share and listen to each other’. Some commented on the inherent value of learning that they are ‘not alone and others have the same problems’. Several talked about how being in the group helped them to ‘open up more’. Participants also told us that they ‘enjoyed learning new skills’ which helped them to cope better with difficulties. There was evidence from some participants that they derived benefit from the group; one person said, ‘I was pleased to come away with coping strategies’, while another said, ‘I used to go out seven times in 22 years and now I can go wherever’. Another said that they found the group, ‘enjoyable and fulfilling’, and some participants talked about seeking further access to psychological therapies elsewhere because the trial had finished.

The third theme was labelled, ‘negative experiences’. Many participants were clear that they wanted to have had longer sessions. One commented, ‘by the time we open up and talked about what bothered us … the group stopped’. Some suggested longer sessions of 90 to 120 min. Others spoke about issues around the dynamics of being in a group, with one participant stating, ‘the group could be easily hijacked’. Participants considered that sometimes their problems were not addressed because other group members talked more. Others felt that some group members spoke about ‘irrelevant issues’ and felt that the therapists should have re-focused the group more frequently. Several spoke about needing more continuity and greater focus on making sure the sessions flowed more effectively, while there were a few participants who commented that they found taking part in a group very difficult and thought the whole experience was negative. However, several commented that they could not think of anything negative about the groups, and several said that the most negative aspect was ‘ending’ and they ‘missed the group’.

The fourth theme was labelled, ‘further adaptations’. Participants described a variety of changes that they would like in order to improve therapy for the future. This included, ‘more preparatory work’ for those who found groups difficult, and several suggested that more individual sessions might motivate some people to change. One person talked about wanting to alternate between blocks of group sessions and individual sessions. Many participants recommended that they would like to see longer sessions in the future which would allow them to consider their problems in ‘more depth, like depression’ and ‘greater work on social skills and friendships’. While several said they really enjoyed homework tasks, some commented that they would like ‘multimedia options, like DVDs, pictures and audio’ for homework and during the sessions.

The final theme to emerge was titled, ‘pragmatic issues’. Participants told us that there were sometimes issues with public transport, travelling, the timings of the group, heating in the rooms and difficulties with parking, all of which they did not like.

### Supplementary analysis

In order to consider whether there may have been a relationship between the number of sessions attended and the outcome, those who had received treatment were split into two subgroups at follow-up; those who had attended <50% of the treatment sessions and those who had attended ≥50% of the treatment sessions. Considering only those participants who had attended ≥50% of the treatment sessions increased the magnitude of difference between the treatment and waiting-list control arms on the primary outcome measure at follow-up 1 than that reported using our per-protocol analysis, although the difference was not statistically significant (Table 5).

**Table 5:** Supplementary analysis using participants who attended < or ≥ 50% of the total treatment sessions prior to Follow-up 1 and 2

	Group		
		
	Treatment arm, mean (s.d.)	Waiting list, mean (s.d.)	Adjustedmean difference^a^ (95% CI), *P*
Hamilton Rating Scale for Anxiety			

Follow-up 1			
<50% sessions	18.2 (6.91), *n*=3	16.3^b^ (7.74), *n*=25	−3.38 (−7.03, 0.27), 0.08
≥50% sessions	14.5 (8.21), *n*=17		

Follow-up 2			
<50% sessions	13.3^b^ (8.57), *n*=22	13.0 (3.46), *n*=3	
≥50% sessions		13.7 (5.64), *n*=20	

a Resulting from an ANCOVA including baseline score.

b Mean calculated using subgroup sample size not split by session attendance.

## Discussion

The conversion rate within this trial was high, and the attrition rate was much lower than that reported within other clinical trials of psychological therapies for anxiety disorders,^26^ suggesting that trials in this area are feasible. Nevertheless, the results indicated that over time, regardless of arm, anxiety symptoms improved significantly. There are likely to be several reasons for this finding; the most likely is that as this is a pilot trial, the probability of making a Type II error had been elevated because of the sample size. Second, it may have been the case that enrolment within the trial led to ‘spontaneous recovery’ amongst those randomised to the waiting list arm. While we did not include a placebo or attention-control condition within this trial, there is evidence that the placebo response has a greater effect within smaller trials.^27^ All of the participants in our trial were told to expect treatment, and for one-half of them, they were told that this treatment would be delayed by 6 months. Over this 6-month period, by instilling a sense of hope and expectation, a placebo response could have occurred, resulting in a reduction in symptoms. Interestingly, Wampold *et al*^28^ reanalysed the data used in a previous meta-analysis investigating placebo effects within trials.^27^ They reported no differences between the effect size associated with the treatment and placebo arms within trials when a disorder was (1) likely to be affected by psychological factors and (2) investigated using a robust methodology. Third, it is important to consider that we did not stop any ongoing or existing treatments for those participants randomised to the waiting list arm. It may have been the case that TAU led to a significant reduction in symptoms for those participants randomised to the waiting list arm. Fourth, participants on average attended 13.6 treatment sessions. It may be the case that participants did not receive a sufficient dose of the intervention, and combined with the sample size, significant treatment effects were therefore not observed. Further, it could also be the case that treatment was not effective. However, all of this must be balanced against the fact that this was a pilot trial, as opposed to a definitive trial, and conclusions regarding treatment effectiveness are therefore premature. Our supplementary analysis suggested that there may be a relationship between the number of treatment sessions attended and outcome, although once again, such a conclusion is highly tentative considering the nature of this pilot trial. It is possible that those who attended <50% of the treatment sessions had greater difficulties with anxiety and found the group intervention more challenging.

The interviews with participants led to a wealth of information about the intervention that is useful for future trials. First and foremost, while a majority of participants reported that they found the intervention useful, and enjoyed attending the groups, they also told us that the sessions were too short. When providing psychological interventions for people who have ASDs, it is important to ensure that participants have sufficient time to engage meaningfully within the intervention, considering their information processing difficulties. Within the context of group-based interventions, therapists need to make sure that they manage and balance the needs of the group, and the needs of individual members, sufficiently. Based on our findings, we would recommend group sessions last at least 2 h. Second, participants made several suggestions for adapting psychological therapies further, which again should be considered by both researchers and clinicians working in this area. Participants indicated they may benefit from more individual sessions, and the suggestion to alternate between blocks of both group and individual sessions might improve treatment efficacy. Such a strategy would allow for greater focus on formulation-driven interventions for clients individually, while at the same time, allowing for any additional therapeutic benefits that may be derived from being part of a group. This would also help to ensure that clients are afforded sufficient time to address their difficulties, something which may take longer for some people with ASDs. Third, participants asked for more innovative homework options, using technology. This may have a positive impact upon engagement. Finally, there were some participants who found taking part in group-based psychological therapy difficult, which appeared to be associated with difficulties with social communication, coupled with marked anxiety problems. It would be important to consider within any future trial whether group-based interventions are appropriate for all participants, and while the aforementioned strategy of alternating between individual and group-based sessions may be helpful, it may be the case that for some people with ASDs, group-based interventions are unlikely to be helpful, considering their difficulties, and such individuals should be offered individual sessions exclusively.

It is important to mention some of the strengths and weaknesses associated with this trial. First, dealing with strengths, the design and methodology were very robust: all of the assessors were masked, and the intervention was standardised. Randomisation and the data were handled independently, while the analysis was undertaken by a statistician masked to subgroup and using the intention-to-treat principle. Participants were drawn from a range of sources and all had a confirmed diagnosis of an ASD along with comorbid problems with anxiety. It was also helpful to have interviewed participants about their views of therapy, providing information to inform a definitive trial. Turning to weaknesses, the current design ensured that all participants received treatment within the context of the research study. Such a design though, as mentioned above, may have led to ‘spontaneous recovery’ within this study. A parallel design, incorporating an attention-control condition, may have been more appropriate and should be considered for definitive trials.

Finally, recently published National Institute for Health and Care Excellence (NICE) guidelines^29^ called for greater support and service planning for those with ASDs, and despite the high prevalence of affective disorders in this population, there are no known definitive trials investigating the efficacy of psychological interventions for this population. The research recommendations made as part of the NICE guidelines^29^ suggested that trials of CBT for people with ASDs needed to consider the delivery method and duration of the intervention, and should test novel treatments in a series of pilot studies, leading to the development of definitive trials. The current study has addressed some of these recommendations, and a large-scale definitive trial, incorporating the changes to treatment as outlined, is now needed to determine whether treatment is effective. We are currently planning such a trial.
